# Preparing the Health Workforce for Digital Antenatal Care: A COM-B-Informed Workshop Using the WHO Digital Adaptation Kit Integration in Cameroon

**DOI:** 10.3390/healthcare14142217

**Published:** 2026-07-21

**Authors:** Miriam Nkangu, Henry Ntanda, Rosemary Tazinya, Mildred Njoache Nkeng, Sarah Ngassa, Valery Ngo, Franck Wanda, Odette Kibu, Fobellah Nkengfac, Ronald Gobina, Tigest Tamrat, Rosemary K. Muliokela, Veronica Shiroya, Alice Tabebot, Ebongo Zacheus

**Affiliations:** 1 Digital and Global Health Lab, Bruyère Health Research Institute, Ottawa, ON K1N 5C8, Canada; 2 Department of Statistics, University of Calgary, Calgary, AB T2N 1N4, Canada; 3 Interdisciplinary School of Health Sciences, University of Ottawa, Ottawa, ON K1N 6N5, Canada; 4 Health Promotion Alliance Cameroon (HPAC), Yaounde BP 17116, Cameroon; 5 The International Center for Research, Teaching and Care (CIRES), Yaounde BP 17116, Cameroon; 6 Faculty of Health Sciences, University of Buea, Buea P.O. Box 63, Cameroon; 7 The Ministry of Public Health, Yaounde P.O. Box 1937, Cameroon; 8 UNDP/UNFPA/UNICEF/World Bank Special Program of Research, Development and Research Training in Human Reproduction (HRP), Department of Sexual, Reproductive, Maternal, Child, Adolescent and Ageing, World Health Organization, CH-1211 Geneva, Switzerland; 9 Centre for Prevention and Digital Health, Medical Faculty Mannheim, Heidelberg University, D-68167 Heidelberg, Germany; 10 Heidelberg Institute of Global Health, University Hospital of Heidelberg, 69120 Heidelberg, Germany

**Keywords:** capacity building, health workforce, digital health, Sub-Saharan Africa, COM-B model, behaviour change, antenatal care guidelines, digital adaptation kit

## Abstract

*Background:* In 2022, we adapted the World Health Organization Digital Adaptation Kit (DAK) for antenatal care (ANC) into the BornFyne digital health platform. Because ANC services in many facilities relied on fragmented paper-based guidance, the DAK represented the first exposure for many providers to a structured, comprehensive ANC guideline framework integrated into a digital system. Understanding providers’ motivation, capabilities, and readiness to adopt guideline-driven digital care was essential to inform implementation strategies. We therefore conducted a workshop to explore healthcare providers’ expectations, perceived challenges, and readiness to integrate a comprehensive digital ANC system into routine practice. *Methods:* A prospective mixed-methods pre–post implementation pilot workshop was conducted between June and August 2023 across four districts. The 5-h workshop targeted ANC providers and included exposure to the WHO ANC DAK and demonstration of its integration into the BornFyne platform. Survey tools assessed perceived knowledge, attitudes, perceived behavioural control, intention, and social norms using Likert scales. Constructs were mapped to the COM-B framework. Paired student’s *t*-tests assessed pre–post changes (*p* < 0.05), and Cohen’s d estimated effect sizes. Qualitative responses were thematically analysed. *Results:* Nineteen participants, including doctors, nurses, midwives, and data managers from public, private, and faith-based facilities, completed the surveys. Most participants were women (90%). Following the workshop, perceived knowledge of behaviour change and digital guideline use significantly increased (t(19) = 3.22, *p* = 0.004, d = 0.78). Although no statistically significant changes were observed in attitudes, perceived behavioural control, intentions, or social norms, descriptive trends suggested improved confidence and openness toward digital ANC systems. Qualitative findings identified increased knowledge as the most important outcome, with 80% of participants emphasizing its role in strengthening capability. Participants also reported greater confidence in integrating digital tools into routine care but highlighted contextual and motivational barriers as ongoing challenges. Acceptability was high: 90% expressed satisfaction, and all participants rated the workshop as useful, relevant, engaging, and worth their time. *Conclusions:* The workshop improved providers’ perceived knowledge and demonstrated high acceptability of DAK-aligned digital ANC systems. While changes in attitudes and behavioural control were not statistically significant, the findings support the feasibility of behaviour change-based digital ANC interventions. Addressing contextual and motivational barriers will be essential for sustained adoption in resource-limited settings.

## 1. Introduction

Digital health is increasingly recognized as a key strategy for strengthening maternal and newborn health services, especially in low- and middle-income countries, where shortages of skilled health workers, fragmented health information systems, and limited access to quality care remain significant challenges. ANC services aim to identify risk factors, prevent and manage pregnancy-related conditions, and promote healthy behaviours during pregnancy and the postnatal period [1,2]. However, implementation gaps continue to affect the quality, continuity, and timeliness of care. Evidence consistently shows that timely, comprehensive, and high-quality ANC is associated with improved maternal and neonatal health outcomes [3,4,5]. Despite these recommendations, the quality of ANC services in sub-Saharan Africa (SSA) remains suboptimal [6,7]. Key challenges span both structural and process-related dimensions of care. Structural barriers include facility readiness limitations, such as inadequate infrastructure, workforce shortages, and suboptimal service delivery models [8]. Process-related barriers include technical challenges, such as poor adherence to clinical guidelines and limited access to skilled care, as well as social factors, including low client satisfaction and engagement [8].

To support high-quality antenatal care, the 2016 World Health Organization (WHO) ANC guidelines recommend that every pregnant woman attend at least eight ANC contacts, beginning in the first trimester and continuing until delivery [1]. To facilitate the translation of the WHO recommendations into interoperable digital systems, the WHO developed the Digital Adaptation Kit (DAK) for antenatal care in 2021 as part of its SMART (Standards-based, Machine-readable, Adaptive, Requirements-based, and Testable) guidelines initiative [9]. The DAK provides standardized digital workflows, core data elements, decision-support algorithms, and functional requirements to enable consistent, high-quality ANC delivery while maintaining alignment with WHO antenatal care recommendations [9]. By providing a common language for clinicians, program managers, implementers, and software developers, DAKs support the development of contextually adapted and interoperable digital health systems and reinforce the use of guidelines at the point of care [9,10,11]. In addition, it includes indicators and metrics that enable monitoring of service quality and effectiveness across levels of the health system [9,10,11]. However, this initiative also underscores the importance of preparing healthcare providers to effectively utilize digital tools and adapt to changing service delivery processes.

In Cameroon, poor adherence to clinical protocols has been identified as one of the major health system challenges in the National Digital Health Strategy [12,13]. This challenge is compounded by the absence or inconsistent availability of ANC guidelines at health facilities [14]. As a result, many providers rely on incomplete documentation tools, contributing to variability in the quality of care and suboptimal maternal health outcomes [15,16,17]. In a prequel to this paper, we described the integration of DAK content into the BornFyne–Prenatal Management System (PNMS) [14], which represented the first comprehensive, standardized ANC guidance in digital format that frontline providers in the four pilot districts were exposed to [14,18]. Following this integration of selected ANC DAK content into the BornFyne-PNMS [14], we conducted a comparative assessment of the DAK content and facility-based ANC registers to identify content gaps, determine the breadth of required data elements, and examine alignment with routine clinical workflows [14]. This assessment indicated that most health care providers had limited prior exposure to a comprehensive ANC guideline comparable to the WHO DAK and minimal experience using digital platforms to support guideline- based care [19]. Building on these findings, we conducted a participatory workshop with health care providers to assess their expectations, perceived motivation, and capability to use a comprehensive, ANC DAK-enabled system, if fully integrated into the routine ANC service delivery system. The workshop was guided by the Capability, Opportunity, Motivation–Behaviour (COM-B) framework [20].

The COM-B framework provides a structured approach to understanding the behavioural determinants of practice change and to identifying areas where targeted support is needed [20]. The model stipulates that behaviour change occurs when individuals possess the necessary knowledge and skills (capability), have a supportive environmental and organizational conditions (opportunity), and are sufficiently motivated to adopt the desired behaviour [20]. Thus, applying the COM-B model to digital ANC implementation can help identify barriers and facilitators to technology adoption while informing capacity-building strategies tailored to frontline healthcare providers. While previous studies have primarily focused on the technological development and effectiveness of digital maternal health interventions, this study uniquely examines health workforce preparedness as a critical determinant of successful digital ANC implementation, using a theory-informed capacity-building approach.

## 2. Methodology

### 2.1. Study Design

A prospective mixed-methods pre–post implementation pilot study was conducted from June to August 2023 to evaluate a 5-h participatory workshop and assess healthcare providers’ perspectives and potential challenges in adopting a DAK–aligned BornFyne-PNMS platform. The workshop was targeted at health professionals engaged in ANC care delivery and was designed to identify and describe providers’ capability, opportunity, and motivational aspects, based on the behaviour model, using the WHO DAK for ANC alongside the BornFyne v2.0 platform as the intervention.

### 2.2. Setting

This pilot project was implemented in four districts in Cameroon. The districts were intentionally selected to ensure the representation of both French (Ayos, Akonolinga) and English (Tiko and Bangem) regions. The two French districts are in rural settings, and the English districts include both rural and semi-urban settings. Given that Cameroon has a heterogeneous health system comprising public, private, and confessional facilities and is a common representation of most countries in sub-Saharan Africa, we ensured that a mix of health facilities was included in this pilot.

In 2017, Cameroon adopted a performance-based financing (PBF) program as a national strategy to improve the quality of care in maternal health services and providers’ performance [21,22]. PBF provides provider incentives based on predefined quality and quantity criteria. However, while PBF improved the structural quality of care (for example, availability of basic equipment and family planning products at health centers), it did not improve the content quality of care for ANC [21,23]. This is evident from our initial observation that antenatal care guidelines were absent from health facilities [14]. Cameroon has a national health management information system, the district health information system (DHIS2), which collects aggregated data on selected maternal health indicators, including those related to antenatal care. Cameroon launched universal health coverage in 2023 with a particular focus on maternal health services and has renewed its national digital health strategy for 2026–2030 to advance its digital transformation agenda [3].

### 2.3. Sampling

The pilot workshop comprised health providers who deliver ANC care at health facilities. They were trained in using the BornFyne application and had all used it for at least 10 months prior to the workshop. A total of 29 participants were trained on the BornFyne platform with selected enhanced DAK elements for ANC. This small sample size was considered appropriate for a pilot implementation study because the primary aim was to assess feasibility, acceptability, provider readiness, and contextual implementation challenges, rather than to generate statistically generalizable estimates.

### 2.4. Framework

The COM-B model involves a behaviour system with three essential interacting conditions, namely, capability, opportunity, and motivation [20].

The individual’s psychological and physical capacity to engage in the activity [20]. This includes having the necessary knowledge and skills to implement the change, which we explored with providers to understand what they consider necessary, given that they have been exposed to the DAK content, partially embedded within BornFyne, and have received training on the DAK-enhanced BornFyne platform. We thought this would give them a better understanding of the expectations and skills needed to deliver the change for ANC from paper to an electronic, DAK-enhanced platform.

The opportunity to engage in change activities encompasses all factors outside the individual that prompt or enable behaviour [20]. In the context of the workshop, we explored, from the providers, what factors they thought they would need or what would need to be changed in their environment to facilitate and ease their use of the platform and adherence to the DAK guidelines for ANC, after being exposed to the BornFyne platform and reviewing the DAK for ANC.

The motivation to engage in change activities involves all the brain processes that energize and direct behaviour, not just goals and conscious decision-making, but also habitual processes, emotional responding, and analytical decision-making [20]. We explored willingness to use the digital application and the DAK guidelines in routine practice.

The rationale for using the COM-B model was grounded in its alignment with the realities of digital transformation, particularly the contextual and structural challenges that influence the implementation of digital platforms in rural settings and across sub-Saharan Africa [24]. First, we identified a notable absence of ANC guidelines at participating health facilities, indicating unsystematic care delivery, as reflected in providers’ responses during training sessions [18]. Some elements are in the official ANC register; some are recorded only in the delivery register; and some are collected informally and documented only in patient-held ANC booklets, depending on individual provider practices. In contrast, core indicators were more consistently captured across facilities. Second, given providers’ limited prior exposure to ANC guidelines, it was essential to explore their perceptions of the DAK content, much of which was new to them, and to assess its full integration into a digital ANC delivery system. Specifically, we sought to (i) identify factors that would facilitate providers’ work if the DAK content were fully integrated into digital platforms such as BornFyne for routine use; and (ii) examine the motivations and challenges providers anticipated following a 10-month exposure to approximately 40% of the DAK-enhanced BornFyne platform. Providers were also encouraged to articulate perceived difficulties associated with potential changes in clinical practice. This workshop served as a pilot study to elicit provider insights and to inform the co-design of a subsequent interventional workshop to strengthen digital competencies and support the delivery of quality ANC. Recognizing both providers’ limited familiarity with digital tools and the infrastructural constraints characteristic of rural settings, this pilot exploratory workshop was considered essential for shaping an effective, contextually appropriate intervention strategy. Furthermore, acknowledging that most participants had not previously been exposed to comprehensive ANC guidelines, a gap that became explicit during training, the workshop was deliberately structured into two complementary components.

### 2.5. Workshop Structure and Design

A participatory, peer-learning workshop was organized by grouping districts with shared language profiles to facilitate experience sharing, peer-to-peer knowledge exchange, and cost efficiency. For the English-speaking districts, providers from Tiko and Bangem were brought together, while providers from Ayos and Akonolinga participated in a joint workshop for the French-speaking districts. Although the English and French workshops were held on different dates, both were held in the same month. This joint format allowed providers to share experiences with the DAK-enhanced BornFyne platform, discuss district-specific challenges, and exchange practical lessons learned from routine implementation. To further support peer learning, the workshops included an additional one-hour targeted support training session to address common challenges identified during pilot implementation and reinforce key digital and clinical workflows. Before the workshops, all participants had used the DAK-enhanced BornFyne platform for 10 months and had been introduced to the ANC DAK components. Given that only about 40% of the DAK elements were integrated into the BornFyne, participants received the DAK data dictionaries and decision-support tools to review beforehand. This pre-distribution ensured a shared baseline understanding and allowed workshop discussions to focus on practical application, adaptation, and improvement rather than initial familiarization. The workshop was divided into two complementary parts.

### 2.6. Workshop Part 1

The first part of the workshop lasted about two hours and focused on introducing and applying the COM-B model to understand health care providers’ readiness for digital transformation. This session focused on the constructs of capability, opportunity, and motivation within the context of changing practice in using the ANC DAK integrated into the digital platform. Since participants had prior experience using BornFyne with DAK integration and had previously participated in exercises comparing DAK content with existing facility-based paper registers [14], this foundational experience was leveraged to support deeper reflection and discussion. The COM-B model was formally introduced, and its core constructs, capability, opportunity, and motivation, were explained and contextualized for digital ANC delivery. Participants were guided to identify the types of support needed to strengthen their skills and improve their ability to use an enhanced, guideline-driven digital platform. In parallel, discussions explored the opportunities and motivational factors required to enable effective adoption and sustained use at scale. To evaluate changes in understanding, participants were asked a series of reflective questions both before and after exposure to the COM-B framework. Finally, the session examined participants’ prior exposure to behaviour change frameworks, theories, or models, and whether they had encountered or applied any such approaches during their professional training.

### 2.7. Workshop Part 2

This second part of the workshop lasted three hours and used the WHO DAK elements for antenatal care as the reference document. In the second component, participants were presented with a scenario in which all DAK elements were fully integrated into the BornFyne platform (or a comparable digital system). Drawing on their experience with the DAK-enhanced BornFyne platform, participants were asked to identify anticipated barriers and challenges they might face in adopting a fully DAK-aligned digital tool for antenatal care delivery. They were also asked to list two reasons that may facilitate or hinder the transition from ANC paper-based records to electronic systems in their settings. Participants were asked to identify internal conditions among health professionals and within their social and physical environments that need to be in place for health providers to adhere to the DAK ANC workflow and guidelines as intended. The session also included an interactive discussion on behaviour change in clinical practice. Participants were encouraged to reflect on their experiences and identify difficulties related to behaviour, attitudes, and routine practices during the shift from paper-based systems to digital platforms. Through this process, participants collectively identified potential barriers to behaviour change, offering valuable insights to guide the design of a subsequent interventional workshop.

### 2.8. Pre- and Post-Assessment of Participants

To assess provider-level change before and after the workshop, we adapted the approach, questions, and Likert scale used by Mullan et al. [25], as listed and described below. Although Mullan et al.’s approach focused on constructs derived from the Theory of Planned Behaviour, including perceived knowledge, attitudes, perceived behavioural control, intention, and social norms [25,26], we retained Mullan et al.’s validated constructs and scales and mapped them onto the COM-B model to strengthen their relevance to implementation and align with behaviour change science. Specifically, we aligned perceived knowledge with psychological capability, reflecting providers’ understanding of behaviour change principles, the integration of digital guidelines (including the DAK guidelines), and confidence in applying them within digital systems. Perceived behavioural control was mapped to both psychological capability and reflective motivation, as it reflects providers’ confidence in their ability to perform ANC tasks in accordance with standard guidelines. Attitudes toward adherence to ANC guidelines were aligned with reflective motivation and categorized into three levels: practice change, willingness to change, and conditional willingness. These levels can be influenced by contextual and institutional factors and reflect beliefs about the value and importance of guideline-concordant practice. The intention to continue using the BornFyne digital platform with embedded DAK components was also categorized as reflective motivation, representing readiness to act. Finally, social norms were mapped onto social opportunity, capturing perceptions of women’s acceptance and reflecting social expectations and perceived approval. For each scale item, providers were asked to provide a rationale for their selected rating. This qualitative explanation enabled a deeper understanding of the quantitative scores and facilitated a broader interpretation of the findings within the relevant theoretical constructs and behaviour change model. This integrated framework allowed us to assess whether provider attitudes and intentions changed following the workshop and to inform broader intervention and behavioural conditions, capability, opportunity, and motivation necessary for sustained adoption of digital ANC with DAK components. By grounding our evaluation in the COM-B framework and adapting previously validated survey tools and scales [25,27], we positioned the findings to inform iterative refinement of interventions and future workshops.

Perceived knowledge: Participants’ perceived knowledge was assessed before and after the workshop using a single item: “On a scale of no understanding to perfect understanding, how would you rate your knowledge of behaviour change constructs of capability, opportunity, and motivation?”

Attitudes 1: Participants were asked with the single phrase “For me, changing my professional practice to use BornFyne-PNMS digital platform that integrates DAK components to enhance adherence to clinical guidelines for antenatal care would be…” Participants were asked to complete this item for 2 attitudes, wisdom and usefulness, with responses on a sliding 5-point Likert scale, ranging from 1 (very wise/very useful) to 5 very unwise/very useless).

Attitude 2: “I am willing and able to change my professional practice to use BornFyne-PNMS as a digital platform that incorporates DAK components to enhance adherence to guidelines for ANC.” 1 = not willing, 5 = very strong will.

Attitude 3: “I am willing and able to change my professional practice to use BornFyne-PNMS as a digital platform that incorporates DAK components to facilitate adherence to clinical guidelines for ANC care, if my health facility decides, starting today, to continue using BornFyne-PNMS for ANC.” 1 = not willing, 5 = very strong will).

Perceived behavioural control: Participants rated the item “I am confident I can change my professional practice to adhere to clinical guidelines for ANC integrated into the BornFyne digital platform” on a 5-point Likert scale, ranging from 1 (strongly disagree) to 5 (strongly agree).

Intention: “I intend to change techniques in delivering ANC care to women over the next coming months.” Participants rated their agreement with the statement on a 5-point Likert scale ranging from 1 (strongly agree) to 5 (strongly disagree).

Social norm: Do you think your patients or pregnant women coming for ANC would prefer and agree that you use a digital platform for their subsequent ANC care delivery? Please rate your response by circling the correct answer that best describes your perception. 1 = Not agree, 2 = agree, 3 = strongly agree).

### 2.9. Workshop Acceptability

This was assessed only at the end of the workshop, and we also adapted Mullan et al.’s approach and scale [25]. Two items were used to assess the acceptability of the workshops, based on the feasibility and acceptability questionnaire developed by Kothe and Mullan [27]. The first set of items asked participants to rate their agreement with each of 7 statements about whether the workshop introducing the COM-B model and the use of the DAK for digital transformation were needed, useful, appropriate for the profession, applicable to their current practices, interesting, exciting, and worth their time. The statements were rated on a 5-point Likert scale, ranging from 1 (completely agree) to 5 (completely disagree). The second set of items asked participants to indicate their satisfaction with the workshop on a 5-point Likert scale, from 1 (completely satisfied) to 5 (completely dissatisfied). The statements were summed to create a total score. A higher score represented greater overall acceptance of the workshop. Higher scores on item 2 represented greater satisfaction with the workshop.

### 2.10. Data Analysis

Descriptive analyses were first conducted to summarize participant characteristics and outcome measures. Continuous variables were reported as means with standard deviations, and categorical variables as counts and percentages. For each variable, the within-participant change from pre- to post-intervention was calculated. The distribution of these change scores was assessed for normality using the Shapiro–Wilk test, which is appropriate for small sample sizes (<50), and was further evaluated through visual inspection of histograms. Based on these assessments, paired Student’s *t*-tests were used as the primary analytic approach to examine within-subject differences, leveraging the correlation between repeated measures to enhance statistical efficiency. Two-sided *p*-values < 0.05 were considered statistically significant. To provide a standardized measure of the magnitude of observed changes, paired Cohen’s d was calculated for each outcome. All analyses were performed using R software, version 4.5.0. Two incomplete responses for pre- and post-surveys were excluded from the analysis.

Participants were asked to respond to survey questions on perceived knowledge, attitudes, perceived behaviour control and social norms. Their responses were then summarized, coded, and qualitatively analyzed to gain a deeper understanding of their survey answers. Finally, participants’ open-ended responses to the two questions were systematically analyzed using a deductive coding approach guided by the COM-B constructs. Participants’ two-point responses on possible challenges related to behaviour change were examined to identify perceived barriers to using a digital platform that fully integrates the DAK components for antenatal care delivery. These responses were then coded and categorized within the corresponding COM-B domains. This approach enabled a structured analysis of providers’ perceived challenges and barriers, directly linking qualitative insights to behavior change constructs.

### 2.11. Ethics Approval

Ethical approval was obtained from the National Ethics Board of Cameroon, ref # 2022/07/1467/CE/CNERSH/SP, and the University of Ottawa Social Science Ethics Board, ref #H-05-22-8077. Administrative clearances were obtained from the Ministry of Public Health at the national level in Cameroon (ref. D30-1440 No. 631-3822) in collaboration with the Division for Health Operations Research (DROS) in Cameroon, the southwest regional delegation of public health (ref. P412/MINSANTE/SWR/RDPH/CB:PF/941/618), and the Central Regional Delegation of Public Health (ref. 1393-4/AAR/MINSANTE/SG/DRSPC). All participants were administered an informed consent form and provided consent to participate.

## 3. Results

**Figure 1:**
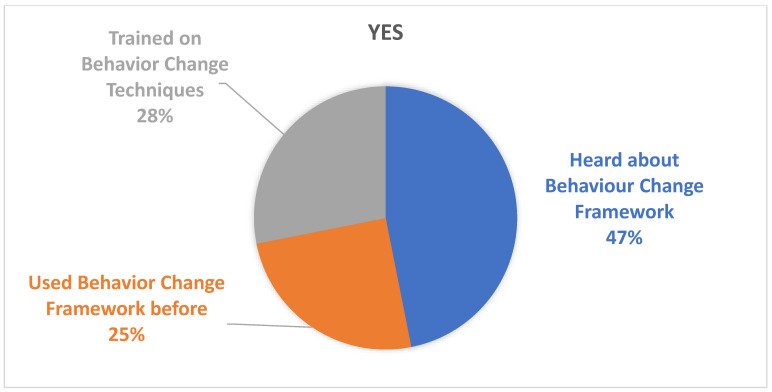
Knowledge about Behaviour Change Framework.

A total of 21 participants attended the workshop. Of the 29 providers who had been trained, were implementing the BornFyne pilot, and had been exposed to the DAK, eight were unable to attend, most of whom were from the French-speaking districts. Most (70%) of the participants have heard of a behaviour framework, but only 38% have used it or been exposed to it, while 42% have received training or attended a workshop on behaviour change (Figure 1). Most participants were women, reflecting the gender distribution of nurses and midwives who predominantly provide antenatal care services. Participants included one medical doctor. The mean age of participants was 34 years (Table 1).

### 3.1. Results of Workshop Part 2

The pre–post assessment results presented in Table 2 summarize outcomes across the constructs analyzed. The quantitative results are presented alongside corresponding qualitative insights, as participants were asked to provide reasons for their scale responses, enabling deeper interpretation of the observed changes.

### 3.2. Perceived Knowledge

A significant increase in perceived knowledge scores from before the workshop (mean = 3, SD = 1.08) to after the workshop (mean = 3.94, SD = 0.73). This represented a change in mean score of 0.94 (95% CI = −1.55, −0.34). This difference was significant (t(18) = 3.31, *p* = 0.004) and had a medium effect size (Cohen’s d = 0.78).

Prior to the workshop, responses from participants perceived knowledge of behaviour change models was limited, fragmented, and largely informal. Most of them described behaviour change in general or intuitive terms, such as counselling clients, personal development, psychology training, or adapting new practices, without reference to any structured model as noted from some participants “behaviour change help to counsel a client to accept a new practice” and “behaviour change technique means teaching someone to change from the way he/she was doing something and adapt a new one”. Following the workshop, participants demonstrated a clear shift toward a structured and applied understanding of behaviour change. Post-workshop responses consistently reflected familiarity with the COM-B framework, with participants explicitly recognizing that behaviour change is not solely motivational but depends on the interaction between skills and knowledge (capability), enabling environments and resources (opportunity), and personal drivers (motivation), as noted by participants that “behaviour change is not only motivational, but opportunities and capability make you change bahaviours”. Another participant nicely put it as “behaviour change depends on the capability availability of the target group, and with the opportunity given and the right motivation, people are willing to adopt healthier and sustainable practice”.

Several participants highlighted that the workshop clarified how behaviour change applies to real-world constraints, including resource availability, training, and system support. Others emphasized increased confidence in applying the framework to their own practice, including supporting colleagues and clients through digital transformation. While a few participants noted the need for additional or continuous training, overall responses indicated a substantial improvement in conceptual clarity, practical relevance, and confidence in using behaviour change principles, as noted, “I now understand the knowledge opportunity and motivation of changing one’s behaviour”; another participant said, “good understanding of behaviour change in the practice of my profession”.

### 3.3. Perceived Behavioral Control

There was no significant change in perceived behaviour control scores from before (mean score = 3.89, SD = 0.9) to after (mean score = 4.06, SD = 1.11) the workshop (t18 = −0.51, *p* = 0.615). This represented a change in mean score of 0.17 (95% CI: −0.85, 0.52), but it was not significant.

Prior to the workshop, providers expressed moderate but cautious confidence in their ability to transition from paper-based registers to digital platforms. Pre-workshop responses emphasized the perceived advantages of digital systems, including easier access to patient information, safer data storage, improved referencing, and the ability to retrieve records beyond the health facility, and participants reported that “the BornFyne-PNMS has some important aspects that are not in our registers”. Providers viewed digital platforms as an innovation that could reduce manual workload, facilitate reporting, enhance continuity of care across providers, and improve communication with pregnant women without requiring physical travel. Despite these perceived benefits, structural and resource-related constraints limited providers’ confidence. Key challenges listed included unreliable electricity, lack of backup power sources, limited access to smartphones and mobile data, and uneven digital literacy among staff. Some providers reported low perceived control over adoption due to these external constraints, noting that confidence remained “not strong” where the cost of data and device access was a barrier, as reported by some participants, “it is good, but we still have some challenges”. Following the workshop, providers reported a marked increase in perceived behavioural control. Post-workshop responses highlighted improved computer skills, greater familiarity with the BornFyne application, and increased confidence in entering and managing detailed antenatal care data, including follow-up visits. Providers emphasized that the peer-to-peer training enhanced their ability to use the platform independently and strengthened their belief that digital documentation is manageable, efficient, and aligned with national and global standards. Post-workshop, digital platforms were increasingly described as easy to manage, time-saving, cost-effective, and practical, with improved adherence to clinical guidelines and more reliable data storage, reducing the risk of data loss “it facilitates adherence to guidelines” and another said that “with BornFyne-PNMS we can strongly follow the guidelines as it facilitates the work when using digital tools”. Providers also expressed greater confidence in maintaining direct client contact and delivering care in a digital environment, viewing the transition as inevitable amid a broader global shift toward digital health systems.

### 3.4. Intention

There was no significant increase in intention scores from before to after the workshop. There was a small difference in scores before (mean score 4.22, SD = 0.94) and after (mean score = 4.06, SD = 1.11) the workshop, with a change in mean score of −0.17 (95% CI = −0.62, 0.95). With more variability in responses after the workshop.

There was no meaningful change in participants’ intention to change how they deliver ANC care in the next month. Their responses indicate that intentions remained stable; at worst, they may have slightly weakened. This suggests participants were more motivated by the DAK-enhanced BornFyne, as they were already familiar with it, but not motivated to translate their readiness or confidence into action with a complete DAK platform. Given that the question referred to a time-sensitive period, they were unsure about certain motivations in the months following the pilot. Most importantly, practical differences were cited, including the continued use of a paper system and uncertainty about whether the health facility is ready to fully use digital tools. Therefore, this decision is not only within providers’ control but also a system-level decision, and it considers other context-specific challenges highlighted in perceived behaviour control.

### 3.5. Social Norms

Before the workshop, providers generally believed that most pregnant women would be receptive to using a digital platform for antenatal care based on their exposure with pregnant women 10 months before the workshop, and particularly because of its perceived benefits, including reduced waiting time, improved follow-up, appointment reminders, and stronger communication with healthcare providers as described in participants as “most of the pregnant women really appreciate the reminders given to them about their next visit. So most pregnant women will be happy to get this reminder”. Participants noted that reminder messages were already well appreciated by many women and helped reinforce attendance at scheduled visits. Digital care was also perceived as appealing due to novelty and growing interest in technology.

However, pre-workshop responses also highlighted important social and structural constraints that could limit acceptance. These included limited access to smartphones, the cost of mobile data, concerns about longer consultation times, and fears that women without smartphones might feel excluded or stigmatized. As a result, providers described women’s acceptance as conditional rather than universal, shaped by access to technology and the broader health system context. Following the workshop, providers articulated a more nuanced and confident understanding of the conditions under which women would agree to digital antenatal care: “they will prefer the digital platform because it will help reduce their waiting time”. Post-workshop responses emphasized that sensitization, counselling, and trust-building are critical to acceptance. Providers reported that when women are clearly informed about benefits such as confidentiality, fewer facility visits, improved continuity of care, and easier communication of concerns, they are more likely to strongly prefer the digital platform, as nicely put by one participant that “because they are informed on time, in real time and are followed up without being displaced”. Participants also highlighted that women’s acceptance is reinforced by positive care experiences, including timely responses to alerts, respectful follow-up, and streamlined access to laboratory services and payments, “because it will be confidential and easier for the health care provider to access the information when necessary”. The workshop strengthened providers’ recognition that digital platforms must be embedded in responsive health system workflows to maintain women’s trust and engagement. Participant said, “ it creates a direct link between patients and doctors”. Despite ongoing concerns about smartphone availability, providers noted that most eligible women encountered during implementation were willing to participate. “Among all those encountered, almost all agreed to participate except those who were excluded and without smartphones,” and “I think women will want to receive information on their next appointment and also have a free platform to get family messages and advice”. Post-workshop responses reflected growing confidence that acceptance would increase as digital health becomes more normalized and sensitization efforts continue, participants said: “it’s new and because many are fascinated about digital technology”. Providers perceived a shift from cautious optimism to stronger social endorsement, with digital antenatal care increasingly viewed as aligned with women’s expectations for quality, convenience, and modern healthcare delivery.

Attitude 1: There was no significant increase in “attitude 1” scores from before to after the workshop. There was a small difference in scores before (mean = 3.68, SD = 1) and after (mean = 4.16, SD = 0.83) the workshop, with a mean change of 0.47 (95% CI = −1.18, 0.23).

Attitude 2: There was no significant increase in “attitude 2” scores from before to after the workshop. However, there was a small difference in scores before (mean = 3.06, SD = 1.06) and after (mean = 3.83, SD = 1.04) the workshop, with a mean change of 0.78 (95% CI: −1.61, 0.05).

Attitude 3: There was no significant increase in “attitude 3” scores from before to after the workshop. There was a small difference in scores before (mean score = 3.26, SD = 1.05) and after (mean score = 4, SD = 1.11) the workshop, with a mean change of 0.74 (95% CI = −1.58, 0.11).

Before the workshop, participants expressed a generally positive but primarily instrumental attitude toward adopting the BornFyne-PNMS platform, as summarized in the following sentence: “If all health facilities were to use BornFyne to care for their clients, all would be touched and would have better care, thus maternal mortality would be reduced to nothing”. Their willingness to change was largely driven by perceived practical benefits, such as improved follow-up of pregnant women, reduced reliance on multiple paper registers, time efficiency, improved client–provider relationships, and better data organization for reporting and statistics; “it helps us cover greater work in less time and helps one relate better to client, therefore I am willing to change”. Participants also associated digital use with improvements in the quality of antenatal care, standardization of services, and potential reductions in maternal mortality. However, this willingness was frequently tempered by concerns about workload, staff shortages, power outages, poor network connectivity, client migration, and limited institutional support, suggesting a conditional rather than fully internalized readiness for change.

Following the workshop, participants’ attitudes shifted toward a more informed, confident, and value-driven willingness to change. Some participant responses included “based on the workshop provided, there are many gaps in antenatal care which I am willing to adopt”; another participant said, “this will improve the quality of care and in the storage of data”. Post-workshop responses emphasized a clearer understanding of how a DAK-enhanced platform supports clinical decision-making, guideline adherence, and continuity of care. Participants increasingly framed their willingness in terms of quality improvement, holistic care, accuracy, safety, and ethical practice, rather than solely in terms of efficiency gains. Many highlighted an enhanced capacity to follow WHO antenatal care guidelines, standardize care delivery, and improve data quality and storage through digital means. This is exemplified in their responses as, “I now understand how this can facilitate my patient or client follow-up”; another participant said, “this will make my work easier and keep my client data regularly,” and another said, “this will increase holistic care of the client and surely reduce maternal mortality”. Importantly, participants demonstrated greater acceptance of the idea that behaviour change is necessary and achievable, recognizing that adopting digital tools requires deliberate shifts in practice and mindset. References to being “in a digital era” reflected the normalization of digital transformation as an expected component of modern healthcare delivery. While some structural concerns (e.g., network and power instability, workload) persisted, they were more clearly articulated as implementation challenges rather than as reasons for resistance.

While participants demonstrated a significant improvement in perceived knowledge following the workshop (*p* = 0.004, d = 0.78), changes in behavioural intention, perceived behavioural control, social influences, and attitudes did not reach statistical significance. This is mainly because baseline scores for intention and attitude measures were already relatively high, suggesting a ceiling effect that limited the magnitude of change detectable after a short workshop intervention. For example, participants reported high pre-workshop intention scores (mean = 4.22/5), indicating a generally positive disposition toward digital ANC due to prior training and exposure to the platform. As a result, substantial improvements in intention may have been difficult to achieve within the study period. Most importantly, the intention question was restricted to a time-sensitive response that largely depended on system-level factors rather than merely the provider’s individual perspective. The moderate effect sizes observed for Attitude 2 (d = 0.47) and Attitude 3 (d = 0.42), despite non-significant *p*-values, suggest that meaningful changes may have occurred but were not detected due to the small sample size. In addition, it also suggests system-level factors that were not directly addressed by the intervention.

Overall, participants reported high satisfaction with the workshop and expressed strong confidence in the training’s relevance, clarity, and practical utility. Many indicated that they had successfully assimilated the knowledge provided and viewed it as directly applicable to improving the quality of antenatal care for pregnant women (Table 3). Participants highlighted that the workshop strengthened their capability to transition from paper-based systems to digital tools, particularly through hands-on practice with the BornFyne platform.

Several noted improved competencies in completing previously challenging fields in the BornFyne application, correcting errors, and conducting patient follow-up more effectively. The use of practical exercises, supported by live data, Wi-Fi connectivity, and mobile devices, was frequently cited as enhancing understanding and confidence. The clarity of presentations and explicit explanations were consistently praised, with participants reporting that previous doubts and challenges were clarified during the sessions. Exposure to digital guidelines and structured workflows improved participants’ understanding of how electronic tools can support adherence to antenatal care standards and reduce documentation errors. Participants also emphasized the broader value of the training in promoting team-based care, noting that digital systems enable multiple providers to access and monitor patient information, thereby improving continuity and accuracy of care. Importantly, the integration of behavior change concepts alongside technical training was perceived as beneficial in strengthening motivation and readiness to adopt digital health tools.

### 3.6. Interactive Discussion on Potential Factors That May Hinder Behaviour Changes in Clinical Practice

*Capability:* Participants’ responses addressed factors that may prevent them from changing their behaviour, as well as those that often deter people from doing so. Participants consistently framed resistance to digital transformation as a capability gap, driven primarily by limited digital literacy, discontinuous training, and cognitive uncertainty. Capability barriers were not solely due to unwillingness but also to structural underinvestment in skill-building. The findings suggest that digital transformation requires strengthening longitudinal capacity rather than one-off training events. We categorized these into three themes as described below.

*Limited digital and computer literacy:* Participants reported that a lack of basic computer skills, low literacy levels, and language barriers can be major constraints on their ability to change their behaviour, which some described as “lack of computer skills”. In addition, most of them mention the fear of the unknown and uncertainty about one’s ability to “comprehend the new system or technology,” which can reduce confidence in using digital tools.

*Insufficient and a need for continuous training:* Participants reported that training should be continuous and sustained, not episodic. Staff turnover without structured onboarding leads to the loss of institutional knowledge. New personnel “not empowered,” undermining continuity of digital practices.

*Cognitive overload and workload pressures:* Overwork and inadequate staffing can reduce providers’ cognitive bandwidth for engaging with new systems, sometimes leading them to view digital tools as adding complexity rather than simplifying workflows.

*Opportunity:* Even when providers were willing and capable, environmental and systemic constraints significantly limited opportunities to adopt digital practices. Opportunity barriers underscore that digital health adoption is not solely an individual behaviour, but a system-level phenomenon. Without infrastructural readiness and institutional alignment, behaviour change interventions risk limited impact. We categorized the response into the following thematic areas.

*Health system and infrastructure constraints:* Power failures, poor internet connectivity, and lack of enabling systems were frequently cited. Participants emphasized that “the means might not be available,” highlighting a misalignment between expectations and facility realities, as one participant nicely noted: “the system put in place may not be in support of the change in behaviour”.

*Organizational and communication barriers:* Poor communication strategies and unclear messaging hindered adoption, as noted by some: “Another problem is the manner in which communication is transmitted.” Systems in place are often not supportive of behavior change, reinforcing paper-based practices.

*Sociocultural and religious influences:* Deeply rooted cultural traditions and religious beliefs were perceived as barriers to change. Some participants felt that no amount of education could overcome entrenched norms. This is exemplified by the words of one participant: “Cultural and traditional aspects of some people, by the nature of the culture and tradition, they will not change their behaviour”.

*Motivation:* Motivational resistance was shaped by habitual practice, psychological inertia, and perceived lack of incentives, rather than outright rejection of digital health. Motivational barriers were both reflective (conscious cost–benefit assessments) and automatic (habits, emotions, identity). This highlights the need for behavior change techniques beyond training, including incentives, social norms, and reinforcement mechanisms. Providers expressed comfort with routine, familiar procedures. Paper-based systems were perceived as easier, faster, and more convenient. Responses were grouped into the following themes:

*Psychological resistance and mindset:* Laziness, ego, pride, and reluctance to learn new ideas was reported by one participant while most reported that behaviour change can be hindered due to static mindsets even in the presence of education or training as referenced “Some people have static mindset that no matter the amount of education, advice or training give to them they will not change” and some described as “unwillingness to leave your comfort zone”, another said “Ego and pride dominate our sense of reasoning”.

*Lack of perceived value or incentives:* Absence of financial or professional incentives reduced motivation. Some participants questioned the benefit of adopting digital tools without tangible rewards. “Some people are very reluctant to learn new ideas, especially when they don’t have any financial benefit”.

*Socioeconomic pressures:* Poverty and personal challenges were reported to potentially influence motivation and openness to change.

**Figure 2:**
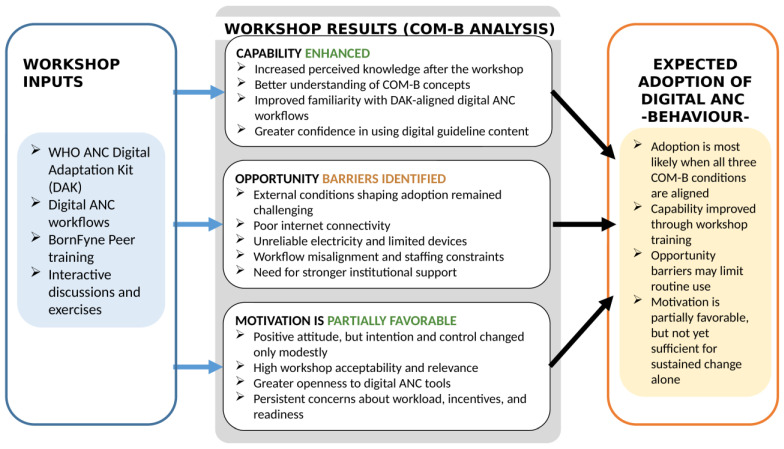
Conceptual flow of the COM-B application adapted to workshop results.

**Conceptual flow of the COM-B application of workshop results:** The conceptual diagram applies the COM-B model to the workshop findings (Figure 2). This indicates that the greatest observed change was in capability (knowledge), while barriers to opportunity and mixed motivation signal the need for additional support strategies. Importantly, the barriers identified under opportunity relate more broadly to digital health implementation challenges overall, rather than exclusively to DAK implementation. However, they provide an overarching understanding of the contextual, infrastructural, workflow, and institutional conditions that may challenge the implementation of digital tools with integrated DAK components. In this sense, the opportunity domain helps situate DAK-enabled implementation within the wider realities of digital transformation in resource-constrained health facilities. The knowledge gained supports readiness, but stronger system supports are needed to sustain behaviour change.

### 3.7. Barriers and Challenges That May Hinder Providers from Changing to Digital Health

We examined the barriers and challenges they might encounter to understand the support needed for a smooth transition from paper to electronic systems and to assess health providers’ readiness. Opportunity-related barriers were the most reported and highlight significant structural and environmental obstacles. Poor internet connectivity, frequent power outages, lack of backup generators, limited access to digital devices, and inadequate workspaces are all reported as possible challenges. Heavy workloads and multitasking across services further constrained their time and focus for digital data entry. Social opportunity barriers were also observed, including limited institutional support, a lack of visible leadership ownership, and inadequate community awareness. In some settings, mistrust about data use and confusion regarding project goals weakened trust and participation. These findings indicate that even when capability exists, digital behaviour change is unlikely without supportive environments. Enhancing infrastructure, aligning workflows, and integrating BornFyne-PNMS into routine facility procedures are crucial to enabling ongoing use. Participant responses revealed interconnected barriers to digital transformation across the three COM-B domains: capability, opportunity, and motivation.

Capability-related barriers mainly involved limited digital literacy, the need for ongoing training, and fear of unfamiliar technologies. Participants pointed out inadequate computer skills, language barriers, and low literacy levels, which were exacerbated by staff turnover and insufficient onboarding for new staff. Heavy workloads and staffing shortages further decreased providers’ capacity to engage thoughtfully with new digital systems.

Opportunity-related barriers highlighted systemic and environmental challenges within health facilities. Participants identified unreliable electricity supply, poor network connectivity, and a lack of adequate digital infrastructure as key obstacles. Organizational issues, such as weak communication strategies and systems misaligned with behaviour change objectives. Additionally, sociocultural and religious norms were seen as limiting openness to new practices, especially where traditional beliefs were deeply rooted.

Motivational barriers were shaped by habitual reliance on traditional paper-based practices, psychological resistance to change, and limited perceived incentives. Participants described comfort with familiar routines, reluctance to learn new approaches, and static mindsets as common challenges. Ego, pride, and laziness were recognised as internal barriers, while the lack of financial or professional incentives diminished motivation to adopt digital tools. Broader socioeconomic pressures, including poverty, can also affect willingness to engage in change.

Overall, these findings corroborate with early lessons from other DAK implementing countries [28], suggesting that successful implementation of digital health solutions, including DAK–enabled systems, requires integrated approaches that concurrently strengthen provider skills, improve enabling conditions within the health system, and address motivational factors through incentives, social influence, and ongoing support.

Common across both districts were poor internet connectivity and unreliable electricity; limited access to smartphones, laptops, and digital infrastructure; and system-level constraints that impede digital workflows. While English-district participants articulated task-specific capability barriers (e.g., workload, language, computer skills). The COM-B analysis revealed both shared and context-specific barriers to the adoption of digital antenatal care across English- and French-speaking districts. Capability constraints were common to both settings, particularly limited digital literacy and confidence among healthcare providers. However, French-district participants more frequently described generalized incapacity and extended digital skill gaps to the broader population, whereas English-district participants identified more task-specific challenges related to workload, computer skills, and understanding of digital workflows (Table 4).

Opportunity barriers differed notably by context. English-district participants emphasized facility-level and operational challenges, including workload burden, misalignment between digital tools and paper registers, and internal communication gaps. In contrast, French-district participants highlighted macro-level structural barriers, including a perceived lack of political will, poverty that limits access to digital devices, and insufficient institutional support for digital transformation.

Motivational barriers also diverged. English-district participants framed resistance to change in terms of cultural norms, static mindsets, and professional identity, suggesting intrinsic and social determinants of motivation. French-district participants, however, emphasized extrinsic drivers, including a lack of material incentives, weak leadership engagement, and economic constraints, indicating that motivation is contingent on system-level support. These findings underscore the importance of contextually tailored behavior change and implementation strategies for scaling digital platforms across diverse district settings. These findings indicate that while providers may recognize the value of digital ANC systems, their psychological and technical capabilities for using them effectively remain uneven. This underscores the need for ongoing, hands-on training and workflow.

## 4. Discussion

The study assessed potential behaviour change among health providers across five constructs, including perceived knowledge, perceived behaviour control, intention, attitude, and social norm, and applied the COM-B model to identify challenges. The findings will inform an interventional workshop to support district-level digital transformation, especially in rural areas. The finding may reflect the distinction between increasing knowledge and achieving readiness for behavioural change. According to the COM-B framework, behaviour is influenced not only by capability (knowledge and skills) but also by opportunity (environmental and organizational factors) and motivation (beliefs, intentions, and attitudes). The workshop was primarily designed to enhance participants’ understanding of digital antenatal care and the DAK, which likely contributed to the observed increase in knowledge. However, behavioural intention and attitudes toward adopting digital ANC may be more strongly influenced by system-level factors that were not directly addressed by the intervention. During discussions, participants identified several potential barriers to implementation, including limited digital infrastructure, unreliable internet connectivity, inadequate equipment, competing clinical demands, and uncertainty regarding organizational support and resource availability. These factors may have constrained participants’ confidence in their responses despite increased knowledge. Future interventions workshop should complement knowledge-building activities with organizational readiness initiatives, infrastructure strengthening, leadership engagement, and ongoing implementation support to address barriers related to opportunity and motivation that influence the adoption of digital ANC in routine practice.

The mixed-methods findings offer important insights into the mechanisms shaping digital ANC preparedness among healthcare providers. Quantitatively, participants showed a significant increase in perceived knowledge after the workshop (*p* = 0.004, d = 0.78), indicating that the training effectively enhanced understanding of digital antenatal care and the use of the COM-B model, including the DAK. In contrast, changes in behavioural intention, perceived behavioural control, social influences, and attitudes were not statistically significant, although moderate effect sizes were observed for some attitude measures, suggesting potential improvements that may not have been detected because of the small sample size. The qualitative findings help explain these further. Participants consistently reported that the workshop increased their awareness of digital ANC workflows, improved their understanding of how digital tools could support clinical decision-making, and enhanced their confidence in navigating the digital system. These findings align with the significant increase observed in the knowledge domain and suggest that the intervention effectively strengthened psychological capability, a key component of the COM-B framework. However, participants also identified several barriers that could limit the adoption of digital ANC in routine practice, including inadequate digital infrastructure, unreliable internet connectivity, limited access to devices, increased workload concerns, insufficient technical support, and uncertainty regarding long-term organizational commitment. Some participants expressed enthusiasm for digital ANC but questioned whether their facilities possessed the necessary resources and system readiness to support implementation. These concerns likely influenced participants’ perceptions of opportunity and behavioural control, thereby limiting changes in intention and attitudes despite increased knowledge. These findings align with previous studies [28] and underscore the importance of complementing workforce training with investments in digital infrastructure, leadership engagement, technical support, and organizational readiness to facilitate the successful implementation and sustained adoption of digital ANC systems.

Participants also suggested increasing the number of practical simulations, including more hands-on exercises on ANC scenarios, and reinforcing the use of questionnaires within the platform. Enhanced interaction among facilitators, healthcare providers, and community health workers was viewed as beneficial and worth strengthening in future sessions. Overall, feedback suggests that future improvements should focus on scaling participation, increasing simulation-based learning, and providing continuous capacity building, rather than on major changes to the workshop structure itself. During provider training, participants emphasized the value of exposure to comprehensive antenatal care guidelines, which they believed would improve the quality of care and adherence to standards [19].

In summary, the workshop was perceived as timely, practical, and empowering, equipping participants with the skills, confidence, and motivation to use digital platforms effectively and deliver higher-quality, guideline-informed antenatal care in an increasingly digital health environment. Participants offered constructive feedback on how to further strengthen the workshop, while many expressed satisfaction with the current format and content. A recurring recommendation was to expand training to include more healthcare providers from different health facilities, emphasizing the need for broader coverage to support consistent platform adoption across the district. Several participants expressed interest in additional and follow-up training sessions, particularly those that use the behaviour change framework and reinforce learning over time. This aligns with findings from other DAK implementation studies from similar contexts [29]. Continuous refresher training was highlighted as important for sustaining skills, especially given staff turnover and the evolving nature of digital competencies. These findings corroborate evidence from other studies across Sub-Saharan Africa, demonstrating that the adoption and sustained use of digital health systems, including electronic medical records (EMRs), are strongly influenced by interacting factors of capability, opportunity, and motivation [28,29,30,31,32,33,34]. Studies from Ethiopia and Nigeria consistently report that limited digital literacy, inadequate training, and insufficient familiarity with electronic systems reduce healthcare providers’ confidence and competence in using digital platforms effectively, reflecting major capability barriers [30,31].

Similarly, organizational and infrastructural constraints, including unreliable electricity, poor internet connectivity, inadequate technical support, limited access to computers, and high workloads, have been identified as critical opportunity-related barriers to integrating digital systems into routine care [28,29,30,31,32,33,34]. Studies by Tolera A et al. and Akwaowo CD et al. found that weak institutional support and resource limitations significantly reduced EMR utilization among health professionals in Ethiopia and Nigeria [30,31]. Likewise, findings from the MatLook project in Cameroon highlighted the challenges of implementing clinical information systems in resource-constrained settings, particularly the need for contextual adaptation, continuous technical support, and workflow integration [31]. They emphasized that digital systems are more likely to succeed when they align with local clinical realities and user needs [31]. Motivational factors also emerged consistently across studies. Providers’ willingness to adopt EMRs was positively associated with perceived usefulness, perceived improvements in service delivery, managerial encouragement, and prior exposure to digital tools [28,29,30,31,32,33,34]. Conversely, resistance to change, low perceived value, and concerns about increased workload reduced motivation to engage with digital systems. Studies conducted in Ethiopia similarly reported that willingness to use EMRs was closely linked to training opportunities, organizational readiness, and positive attitudes toward technology [33,34].

### 4.1. Implication for Policy and Practice

At the individual and facility levels, healthcare providers reported staff reluctance to transition from paper-based registers to digital systems, driven by limited digital skills, computer illiteracy, and discomfort with new technologies. These challenges were compounded by inadequate staffing, multitasking across service areas (ANC wards, labour rooms, and administrative duties), and facility congestion, which limited time and space for digital data entry. At the system level, unreliable internet connectivity, frequent power outages, lack of backup electricity, limited access to computers and smartphones, and poor road networks, particularly in rural settings such as Bangem, were major constraints. These infrastructural limitations disrupted real-time data entry, delayed alerts, and reduced confidence in the platform’s functionality. Together, these findings indicate that provider readiness cannot be separated from broader health system readiness; successful adoption of digital ANC tools depends not only on individual willingness or knowledge, but also on whether facilities have the infrastructure, leadership support, workflow alignment, staffing capacity, and technical assistance needed to sustain use. These findings reinforce the dynamic interaction proposed within the COM-B framework [20]. Limited capability restricts providers’ confidence and competence; constrained opportunities undermine the practical feasibility of using digital systems; and weak motivation reduces willingness to change established practices. Importantly, these domains are interdependent: high workload and poor infrastructure can erode motivation, while low motivation may reduce engagement in training opportunities that could strengthen capability. Therefore, implementation strategies should move beyond one-time training and include integrated readiness-building activities, such as continuous digital competency development, facility-level workflow redesign, infrastructure strengthening, leadership engagement, supportive supervision, and feedback mechanisms that reinforce routine use. These findings underscore the importance of integrated implementation strategies that simultaneously address technical capacity building, supportive organizational environments, and behavioural engagement to support successful digital health adoption in most African health systems.

### 4.2. Strengths and Limitations

This study employed a theory-informed design guided by the COM-B model, enhancing its conceptual rigour and ensuring a systematic assessment of capability, motivation, and opportunity [20]. The mixed-methods pre–post approach enabled triangulation of quantitative changes with qualitative insights, offering a detailed understanding of provider readiness for digital guideline adoption. The use of previously validated tools and scales [25,27]. Including participants from public, private, and Faith-based facilities, and from French and English districts, with a mix of nurses, midwives, and medical doctors, improved contextual relevance and transferability. High acceptability and engagement during workshops further support the feasibility of scaling up the workshop. The study also addressed an important implementation gap: limited exposure to comprehensive ANC guidelines in a real-world rural health system setting. However, the study had some important limitations. Given that this was a pilot study, the small sample size reduces statistical power and limits the generalizability of the findings. The short-term assessment prevented evaluation of long-term behavioural change or of the digital platform’s actual use in practice. Self-reported measures may be influenced by social desirability bias. Additionally, infrastructural issues such as connectivity and device access were not objectively assessed, thereby limiting understanding of structural barriers to opportunity. However, the findings provide insights that will inform subsequent co-design interventional workshops.

## 5. Conclusions

The workshop significantly improved providers’ perceived knowledge and demonstrated high acceptability, indicating readiness to engage with DAK-aligned digital ANC systems. While attitudes and perceived behavioral control showed positive but non-significant shifts, contextual and motivational barriers remain critical to address. These findings support the feasibility of a behaviour–change–informed, digitally integrated ANC intervention and highlight the need for targeted strategies to strengthen motivation, increase opportunities, and promote sustained adoption in resource-constrained settings.

## Figures and Tables

**Table 1:** Participant demographic characteristics.

Variable	Frequency	Percentages
*Sex*		
Female	19	90.48
Male	2	9.52
*District*		
Akonolinga	1	4.76
Ayos	3	14.29
Bangem	7	33.33
Tiko	10	47.62
*Professional*		
Doctor	1	4.76
Data manager	1	4.76
Midwife	9	42.86
Nurse	10	47.62
*Facility Type*		
Private for profit	5	25
Public	14	70
Confessional	1	5
*Number of years in service*		
1	3	15
3	3	15
4	1	5
5	3	15
6	2	10
8	3	15
11 or more	5	25

Participants’ Age Mean [SD], 34.28 [7.45], Min 21, Max 49.

**Table 2:** Within-group comparisons pre- to post-test.

Variable n	Pre-Test Mean (SD)	Post-Test Mean (SD)	t-Value	*p*-Value	d
Perceived Knowledge 18	3 (1.08)	3.94 (0.73)	−3.31	0.004	0.78
Perceived Behavioral Control 18	3.89 (0.9)	4.06 (1.11)	−0.51	0.615	0.12
Intention 18	4.22 (0.94)	4.06 (1.16)	0.45	0.660	−0.11
Social 19	2.21 (0.42)	2.32 (0.58)	−0.62	0.541	0.14
Attitude 1 19	3.68 (1)	4.16 (0.83)	−1.41	0.173	0.32
Attitude 2 18	3.06 (1.06)	3.83 (1.04)	−1.98	0.063	0.47
Attitude 3 19	3.26 (1.05)	4 (1.11)	−1.83	0.084	0.42

**Table 3:** Workshop acceptability.

Acceptability Constructs	Frequency	Percentage	Frequency (%)
*Needed*			
Strongly agree	11	57.9	11 (57.9%)
Agree	7	36.8	7 (36.8%)
Completely disagree	1	5.3	1 (5.3%)
*Useful*			
Strongly agree	13	68.4	13 (68.4%)
Agree	5	26.3	5 (26.3%)
Completely disagree	1	5.3	1 (5.3%)
*Appropriate for the profession*			
Strongly agree	12	63.2	12 (63.2%)
Agree	6	31.6	6 (31.6%)
Completely disagree	1	5.3	1 (5.3%)
*Applicable to my current practices*			
Strongly agree	7	36.8	7 (36.8%)
Agree	11	57.9	11 (57.9%)
Completely disagree	1	5.3	1 (5.3%)
*Interesting*			
Strongly agree	13	68.4	13 (68.4%)
Agree	5	26.3	5 (26.3%)
Completely disagree	1	5.3	1 (5.3%)
*Exciting*			
Strongly agree	7	36.8	7 (36.8%)
Agree	10	52.6	10 (52.6%)
Disagree	1	5.3	1 (5.3%)
*Worth your time*			
Strongly agree	11	57.9	11 (57.9%)
Agree	6	31.6	6 (31.6%)
Completely disagree	1	5.3	1 (5.3%)
*Satisfied with the workshop*			
Strongly Agree	4	21.1	4 (21.1%)
Agree	4	21.1	4 (21.1%)

**Table 4:** Key differences that emerged by district and equity considerations.

COM-B Domain	English-Speaking Districts	French-Speaking Districts
Capability	Task-specific digital skills, workload, fear of complexity	Generalized incapacity, community-level digital gaps
Opportunity	Workflow misalignment, staffing, facility constraints	Political will, poverty, infrastructure, leadership support
Motivation	Cultural norms, mindset, identity, resistance to change	Incentives, material support, institutional commitment

## Data Availability

All data collected have been published in this paper.
